# Zika Virus-Specific IgY Results Are Therapeutic Following a Lethal Zika Virus Challenge without Inducing Antibody-Dependent Enhancement

**DOI:** 10.3390/v11030301

**Published:** 2019-03-26

**Authors:** Kyle L. O’Donnell, Bernadette Meberg, James Schiltz, Matthew L. Nilles, David S. Bradley

**Affiliations:** 1 Department of Biomedical Sciences, University of North Dakota, School of Medicine and Health Sciences, Grand Forks, ND 58202-9037, USA; kyle.odonnell@und.edu (K.L.O.); bernadette.meberg@und.edu (B.M.); matthew.nilles@und.edu (M.L.N.); 2 Avianax, LLC, Grand Forks, North Dakota, ND 58202, USA; jschiltz@schiltzfoods.com

**Keywords:** Zika virus, IgY, Immunotherapy

## Abstract

The Zika virus (ZIKV) is a newly emerged pathogen in the Western hemisphere. It was declared a global health emergency by the World Health Organization in 2016. There have been 223,477 confirmed cases, including 3720 congenital syndrome cases since 2015. ZIKV infection symptoms range from asymptomatic to Gullain–Barré syndrome and extensive neuropathology in infected fetuses. Passive and active vaccines have been unsuccessful in the protection from or the treatment of flaviviral infections due to antibody-dependent enhancement (ADE). ADE causes an increased viral load due to an increased monocyte opsonization by non-neutralizing, low-avidity antibodies from a previous dengue virus (DENV) infection or from a previous exposure to ZIKV. We have previously demonstrated that polyclonal avian IgY generated against whole-killed DENV-2 ameliorates DENV infection in mice while not inducing ADE. This is likely due to the inability of the Fc portion of IgY to bind to mammalian Fc receptors. We have shown here that ZIKV oligoclonal IgY is able to neutralize the virus in vitro and in IFNAR^−/−^ mice. The concentration of ZIKV-specific IgY yielding 50% neutralization (NT_50_) was 25 µg/mL. The exposure of the ZIKV, prior to culture with ZIKV-specific IgY or 4G2 flavivirus-enveloped IgG, demonstrated that the ZIKV-specific IgY does not induce ADE. ZIKV IgY was protective in vivo when administered following a lethal ZIKV challenge in 3-week-old IFNAR^−/−^ mice. We propose polyclonal ZIKV-specific IgY may provide a viable passive immunotherapy for a ZIKV infection without inducing ADE.

## 1. Introduction

Flaviviruses are a major health concern throughout the world. A newly emerged flavivirus that has major health implications is the Zika virus (ZIKV). A majority of ZIKV infections are asymptomatic in the host. Severe ZIKV symptoms extend to neurological diseases including Gullian–Barre Syndrome (GBS) [1,2] and congenital Zika syndrome (CZS). CZS can be further differentiated into microcephaly, brain abnormalities, and other severe birth defects [3,4,5,6,7]. From February 1 to November 18, 2016, the world health organization (WHO) declared ZIKV a world health threat and pushed for the development of vaccines and antivirals to combat ZIKV infection [8]. 

ZIKV is a member of the *Flaviviridae* family, which also includes the dengue virus (DENV), West Nile virus (WENV), Japanese encephalitis virus (JEV), yellow fever virus (YFV), and tick-borne encephalitis virus (TBEV). ZIKV is primarily spread by the *Aedes* species of mosquitoes [9]. Recently, our lab has identified that *Aedes vexans* is also a potential competent vector which extends the geographical range of infection from tropical to temperate climates [10]. In the absence of a quality vector control in the countries affected by ZIKV, the development of new antivirals and vaccine candidates is required to control the spread of ZIKV. 

A severe flavivirus infection can be attributed to cross-reactive inflammatory T cells and non-neutralizing antibodies that induce antibody-dependent enhancement (ADE) [11,12]. ADE occurs via antibodies from a primary infection binding to a heterotypic flavivirus. The viral immune-complex is then recognized by the FcγR on a dendritic cell or macrophage and binds via the Fc portion of the antibody. This complex is then internalized in the endosome where the pH decreases as the endosome matures. This drop in pH lowers the affinity of the antibody and virus, allowing the virus to dissociate from the antibody. Once the virus has dissociated from the antibody, normal viral replication is commenced by the fusion of the envelope to the membrane of the endosome releasing the viral nucleic acid [12]. The titer present from the primary flaviviral infection directly influences ADE. A high titer from the primary infection can result in the protection from cross neutralizing antibodies. A lower titer has been shown to indicate the risk of ADE in a human cohort study [11].

To date there is an unmet need for an effective antiviral therapy for ZIKV infection. Recently, one area of research that is showing promising results is the utilization of truncated antibodies that lack the Fc portion. Settler et al. demonstrated that truncated monoclonal antibodies were able to neutralize a primary ZIKV infection and a secondary DENV infection without inducing ADE. They further go on to demonstrate that the truncated monoclonal antibodies were able to protect IFNAR^−/−^ mice challenged with a lethal dose of ZIKV [13]. We hypothesize that avian IgY, the avian homolog of IgG, will be an effective therapeutic antibody against flavivirus infections based on the unique characteristic that full length IgY does not bind to mammalian FcγR [14]. The intrinsic characteristics of IgY ablate the need for genetic manipulation during antibody production as seen in Settler et al. Previously we have demonstrated that Dengue-specific IgY was effective at neutralizing lethal infections with DENV2 without inducing ADE [15]. 

IgY is the avian homologue of mammalian IgG and shares characteristics with mammalian IgG and IgE. IgY is the predominant isotype in sera after the initial production of IgM and is the primary antibody produced upon a secondary response [16,17]. IgY is found in two isoforms in the serum of water fowl: full-length IgY that contains two constant regions and an alternatively spliced IgY that lacks these two constant regions [17]. The alternatively spliced IgY would be the avian structural equivalent of the truncated IgG proposed by Settler et al. Previous studies from our laboratory have demonstrated the efficacy of the utilization of IgY in viral infections. We first demonstrated that Andes virus-specific IgY provided protection from Hantavirus pulmonary syndrome in a hamster model [18]. Most recently, we have demonstrated that DENV-specific IgY was able to protect AG129 mice from a lethal dose of DENV2 [13]. 

There are many benefits for the utilization of IgY as an immunotherapy in mammals; first the phylogenic distance between the avian species producing the antibody and the recipient mammalian host allows for a much broader range of antigenic recognition. This is due to the increased avidity for mammalian antigens and the ability to recognize antigens that normally would not be immunogenic in a mammalian host. The increased antigenic recognition and avidity of IgY are attributed to the unique antibody maturation process. During IgY maturation, gene hyper conversion allows for pseudo-V genes to recombine with the existing variable regions of the Ig genes to produce mature B-cells. The second reason is V-J flexible joining; this is when the partial conversions of rearranged segments are replaced by pseudo-V(D) genes. The third mechanism to produce diversity is the large amount of somatic hyper mutations that is undergone [17,19,20,21]. Another critical characteristic of IgY is the lack of interaction with mammalian or known bacterial FcγR or Fc binding receptors. The lack of interaction with Fc receptors leads to the premise that IgY will not induce ADE when encountering subsequent serotypes of Dengue or cross-reactivity with ZIKV. Further, IgY does not interact with mammalian complement systems, which, in the case of a DENV infection, is a positive attribute. A study demonstrated that patients with Dengue hemorrhagic fever had elevated levels of activated complement components, specifically C3a and C5a [22]. It has been demonstrated that antibodies binding to DENV NS1 attached to endothelial cells activated the complement cascade leading to the lysis of non-virally infected cells [23,24]. Not only are the immune characteristics of IgY attractive but also the industrial production potential makes IgY an attractive therapeutic candidate. Due to the fact that IgY can be isolated from the eggs of laying geese or hens, this allows for a noninvasive way of harvesting an immunotherapy compared to production in a mammalian host, such as rabbits. The antibody yield is also much greater per month due to this noninvasive aspect; 1300 to 1500 mg of IgY can be produced from one bird per month compared to 200 mg from a mammalian source [16]. There are currently large-scale production methods and sites functional at this time, making IgY an economically feasible therapeutic. 

In this study we demonstrate the efficacy of goose-derived anti-ZIKV IgY in vitro and in vivo. Specifically, ZIKV IgY produces a protection greater than a 50% reduction in a plaque reduction neutralization test, and ZIKV IgY did not produce ADE in vitro. Finally, we demonstrate that ZIKV-specific IgY can protect 3-week-old IFNAR^−/−^ mice from a lethal ZIKV challenge. 

## 2. Materials and Methods

### 2.1. Ethics Statement

The research was conducted in compliance with the Animal Welfare Act and adheres to the recommendations in the Guide for the Care and Use of Laboratory Animals of the National Institutes of Health. The protocol was approved by the Institutional Animal Care and Use Committee at the University of North Dakota (project number: 1303) and the Institutional Biosafety Committee (project number: 201802-012). 

### 2.2. Geese

The geese (*Anser domesticus*; 25 months old) used to produce IgY are an inbred hybrid of a combination of German Embden, the Royal Chinese, and the Royal English breeds. The animals were housed at the Specific Pathogen Free facility on the Schiltz Goose Farm, Inc. The barn also housed a clean room for international organization of standardization egg production, which was equipped with high efficiency particulate air filtration to remove circulating particles. Only authorized personnel were allowed into the facility. 

### 2.3. Mice

B6.129S2-Ifnar1^tm1Agt^/Mmjax (IFNAR^−/−^) were purchased from Jackson Laboratory MMRRC stock #32045 [25]. The mice were maintained in clean rooms in the Center for Biomedical Research Center, UND. 

### 2.4. Viruses and Cell Lines

The Zika virus PRVABC59 was propagated in Vero cells (American Type Culture Collection). Briefly, the Vero cells at 80% confluency in 6-well plates were infected at an multiplicity of infection (MOI) of 0.1 and allowed to adhere for one hour at 37 °C with 5% CO_2_. The cells were washed with DMEM, containing 10% fetal bovine serum (FBS) (Atlanta biologicals, Atlanta, GA, USA), penicillin/streptomycin (Corning, Corning, NY, USA), and 2.5 mM HEPES, (cDMEM). Three ml of cDMEM was replaced in the wells; the plates were incubated at 37 °C with 5% CO_2_ for five days. The cell supernatant was centrifuged at 4000× *g* for 5 min at 4 °C to remove cell debris. The clarified cell supernatant was diluted 1:2 in heat inactivated FBS and frozen at −80 °C. The resulting viral stocks were quantified via plaque assay. 

### 2.5. Immunization

The immunization schedule to produce DENV-specific IgY was previously described. The Zika virus PRVABC59 was propagated in Vero cells to a titer of 3.8 × 10^5^ plaque forming unit (PFU)/mL. The cell supernatant was centrifuged at 4000× *g* for 10 min at 4 °C to remove cell debris. The resulting supernatant was exposed to 5.23 Mrad of gamma irradiation. The resulting viral stock was shown to be inactivated via plaque assay quantification. The geese were vaccinated with 1.52 × 10^5^ PFU/mL of irradiated Zika virus. The dose was based on a pre-irradiated viral titer. Immunizations consisted of 2 × 200 µL subcutaneous injections at the back of the neck in two different injection spots. The eggs were collected starting from week 6 after the first vaccination and stored at 4 °C until further use.

### 2.6. Purification of IgY from Goose Egg Yolk

Zika virus IgY was purified using polyethylene glycol. Briefly, the egg yolk homogenate was thawed and centrifuged at 10,000× *g* for 30 min at 4 °C. The supernatant was passed through a 0.2 µm sterile filter and concentrated using a 100 kDa (MilliporeSigma, Burlington, MA) tangential flow unit. Twelve percent (weight/volume) of 6000 MW polyethylene glycol (PEG) was added to the concentrated supernatant that was then stirred for 30 min at room temperature. The PEG mixture was then centrifuged at 10,000× *g* for 30 min at 4 °C. The supernatant was discarded, and the pellet was resuspended to the original volume in 1× phosphate buffered saline (PBS). Twelve percent 6000 MW PEG was then added to the resuspended pellet and mixed for 30 min at room temperature. The mixture was centrifuged at 10,000× *g* for 30 min at 4 °C, and the pellet was saved. The pellet was resuspended in the original volume of 1× PBS (pH 7.4) and concentrated to 50 mL using a 100 kDa cassette (MilliporeSigma); the final sample was then diluted with 450 mL of 1× PBS. The samples were additionally dialyzed in 1× PBS to remove residual PEG. Individual eggs were pooled together to create a lot of oligoclonal anti-ZIKV IgY. 

### 2.7. Antibody Detection

The binding activity of flavivirus IgY was determined by ELISA in three independent experiments in triplicate as previously described [17]. Briefly, 100 µL of antigen was coated onto a 96-well microplate and stored overnight at 4 °C. The following day, the plate was washed 3× and then blocked with 400 µL of a blocking buffer and incubated at room temperature for 30 min. The wells were washed three times and incubated with 100 µL of IgY serially diluted 1:2 across the wells of the plate. The plate was incubated at 37 °C for 30 min. The plate was washed 3× and blocked at room temperature for 10 min. One hundred µl of biotinylated rabbit anti-goose IgY antibody in a blocking buffer was added to the plate and incubated at 37 °C for 30 min. Following this, 100 µL of a diluted strepavidin-horseradish peroxidase antibody in a blocking buffer was added to each well, and the plates were incubated at 37 °C for 30 min. Finally, the plate was washed 3×, and 100 µL of o-phenylenediamine dihydrochloride color substrate was added to each well and developed at room temperature protected from light for 15 min. The reaction was stopped by adding 50 µL of 1 N H_2_SO_4_, and the absorbance was read in a BioTek plate reader at A_490_. The data are represented as endpoint titers. 

### 2.8. Plaque Assay

Vero cells were seeded into 6-well plates at 2 × 10^5^ cells/well and allowed to come to confluency. The viral stocks were thawed in a 37 °C water bath and diluted from 10^−1^ to 10^−7^ in cDMEM. Triplicate wells were infected with 250 µL of the viral dilutions, whereas the cell controls were inoculated with the media alone. The plates were placed at 37 °C with 5% CO_2_ for 1 h, lightly shaking every 15 min. The viral inoculum was removed, and the wells were washed with 1× PBS. Next, 3 mL of 1:1 of the 2% methyl cellulose and cDMEM mixture was added to each well. The plates were incubated for 7 days (ZIKV) and 10 days (DENV). At the end of the incubation, the overlay was removed and the cells were fixed with 400 µL of 10% formalin for 30 min at room temperature. Formalin was removed, and a crystal violet solution was added to each well for 10 min to stain the cell layer. The crystal violet was removed, and the wells were washed 3–5× with 1× PBS until the plaques were clearly visible. 

### 2.9. Plaque Reduction Neutralization

The plaque reduction neutralization assay (PRNT) is similar to the plaque assay noted above with the following exceptions. The virus is diluted to 40–60 PFUs and is then treated with IgY ranging in concentrations from 250 µg/mL to 2.5 µg/mL (*n* = 9). Plaques are then counted, and a 50% or greater reduction of plaques was considered a significant reduction (in accordance with WHO standards) [25].

### 2.10. Antibody-Dependent Enhancement

The presence of ADE was assessed using the protocol in Diamond et al. with minor modifications [26]. We used an MOI of 2 for ZIKV and 10 for DENV due to the difference in binding kinetics between ZIKV and DENV noted in Charles and Christofferson [27]. The THP-1 cells were seeded at 1.76 × 10^4^ cells/well for the DENV infection and 1.2 × 10^5^ cells/well for ZIKV to accommodate for differences in the titer of the viral stocks. The antibody treatment was done with 300 ng of mAb 4G2 (Anti-flavivirus group antigen antibody, Novus); 1 µg and 100 µg of anti-ZIKV IgY were incubated at 37 °C with 5% CO_2_ for 1 h prior to infection. The cells were infected for 90 min at 37 °C with 5% CO_2_ (*n* = 9). After infection, the cells were centrifuged at 900× *g* for 5 min and washed 3×. The cells were suspended in cDMEM. Identical concentrations of the treatment antibody were added to the cells and incubated for four days at 37 °C with 5% CO_2_. The cells were centrifuged again, the supernatant was collected, and the viral titer was determined by a plaque assay in triplicate. ADE was also assessed by co-incubating virus and antibody solutions for 1 h at 37 °C with 5% CO_2_ and then added to K562 cells and incubated for 1 h under the same conditions (*n* = 6). The cells were then washed with 100 µL of cDMEM and maintained for 2 days at 37 °C with 5% CO_2_. The cells were fixed with 10% formalin for 30 min at room temperature. The cells were subsequently washed with an FACS buffer, permeablized with a flow cytometry perm buffer (TONBO, San Diego, CA, USA), and stained with a 4G2 Alexa 488 conjugated antibody (Novus). Once stained for 2 h in the dark and washed, the cells were analyzed via FACS and the number of Alexa 488 positive cells were calculated [28].

### 2.11. In Vivo ZIKV-Specific IgY Protection

The therapeutic potential of ZIKV-specific IgY was tested using conditions that cause 100% mortality in IFNAR^−/−^ mice. Three-week-old IFNRA^−/−^ mice were challenged with a lethal dose of ZIKV PRVABC59 (1.0 × 10^4^ PFUs) intravenously (i.v.) via the retro-orbital sinus. Twenty-four hours after infection, the mice were injected intraperitoneally (i.p.) with 500 µg of ZIKV-specific IgY (*n* = 6) or the control naïve IgY (*n* = 8) in a volume of 100 μL of PBS, or with 100 μL of PBS alone (*n* = 6) as a negative control. Twenty-four and 48 h after infection, the mice were injected intraperitoneally with 500 µg and 1 mg of ZIKV-specific IgY (*n* = 9 and 6 respectively) or the control naïve IgY (*n* = 9 and 6 respectively). The mice were observed twice daily for 14 days for signs of morbidity and mortality.

### 2.12. Viral Load Quantification

At the mortality end point or conclusion of the study, the tissue samples (brain, spleen, and liver) were processed for viral load. Briefly, the tissues were collected in 1 mL of cDMEM and homogenized using a bullet blender. The supernatant was then centrifuged at 4500× *g* for 10 min. The clarified supernatant was then diluted in cDMEM in order to allow for 20–50 plaques per well in a 6-well plate. The plaque assay protocol described above was then used to quantify the viral load in the respective organs. The viral copy number in the respective organs was also quantified via RT-qPCR. Viral RNA was extracted from the clarified tissue supernatant with the Viral RNA mini kit (Qiagen, Hilden, Germany). We then utilized the Zika virus Genesig Easy kit (Genesig, Plymouth Meeting, MA) to quantify the viral copy number of the infected organs. 

### 2.13. Epitope Mapping

Anti-ZIKV IgY epitopes were mapped using peptide arrays for E, prM, C, NS1, NS2a, NS3, and NS5 proteins. Eleven amino acids overlapping 15 mer peptides were covalently linked to a microarray slide (JPT Innovative Peptide Solutions, Mainz, Germany), according to the manufacturer’s protocol. Briefly, an uncoated slide and the slide containing the microarray were gently laid upon one another, separated by spacers. Fifty µg/mL of the control naïve IgY or anti-ZIKV specific IgY was incubated on the slides overnight at 4 °C in a moist environment. The slides were then rinsed 5× with tris-buffered saline and Tween 20 (T-TBS) for 4 min and then 5× for 4 min with 18.5-ohm water. The slides were then incubated with 1 µg/mL of a Cy5 goat anti-chicken IgY secondary antibody (Abcam, Cambridge, UK) for 45 min protected from light. The slides were washed 5× with T-TBS and 5× with ultrapure water and dried with dust-free, oil-free, canned air. A Genepix 4000 microarray reader was used to measure the fluorescent signal at a pixel size of 10 µm. The signal intensity mean values were calculated for each of the 3 sub-arrays and corrected for background. 

### 2.14. Statistical Analysis

Differences in PFU/mL and copy number between the antibody groups and controls as well as the comparison of organ weights between groups were analyzed using ANOVA with a Bonferroni posttest for multiple comparisons. The survival was measured using the Kaplan Meir and Mantel–Cox tests. The Mann–Whitney test was used to compare the difference in body weight percentages. GraphPad Prism (Version 7.0a, GraphPad Sofware Inc., CA, USA) was used for a statistical analysis with *p*-values of <0.05 considered significant. 

## 3. Results

### 3.1. Antibody Characterization

**Figure 1:**
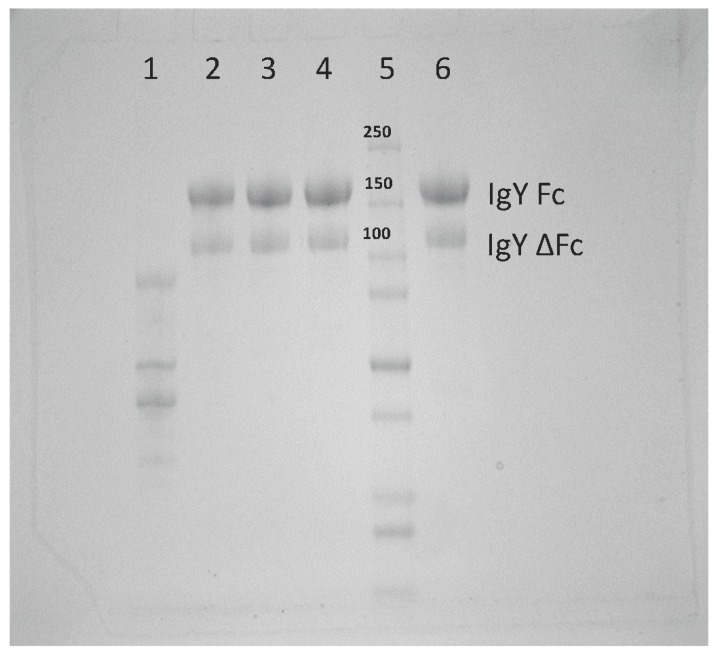
Anti-ZIKV (Zika virus) IgY purification: The purity of anti-ZIKV IgY after the second polyethylene glycol (PEG) precipitation. Lane 1, supernatant from the PEG precipitation; lane 2, resuspended PEG pellet; Lanes 3 and 4, molecular weight concentration and diafiltration; Lane 5, molecular weight marker; and Lane 6, control naïve IgY that was previously purified via mercapto-ethyl pyridine HyperCel Hydrophobic Charged Induced Chromatograph for comparison.

The characterization of polyclonal anti-DENV2 has been demonstrated previously by our lab [14]. Following the polyethylene glycol purification of anti-ZIKV IgY, SDS-PAGE was used to verify the presence and purity of full-length IgY and alternatively spliced IgY. We were able to detect both the full-length and alternatively spliced forms of anti-ZIKV IgY from the goose egg yolk homogenate (Figure 1). An ELISA was then performed to detect the presence of ZIKV-specific IgY and to determine the endpoint titer of our IgY preparation. The mean endpoint titer of the samples tested was 1:160,000. 

### 3.2. In Vitro Viral Neutralization and Enhancement of Zika-Specific IgY

**Figure 2:**
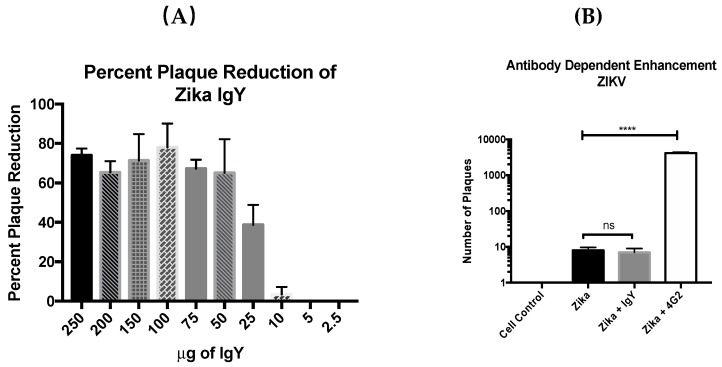

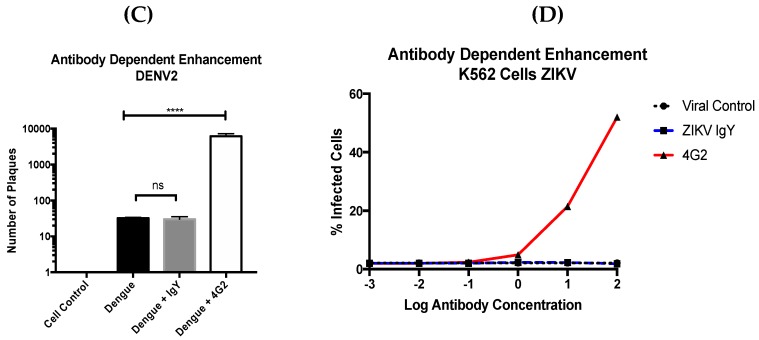
Anti-ZIKV IgY neutralizes ZIKV in vitro without antibody-dependent enhancement. (**A**) Anti-ZIKV IgY neutralized the ZIKV infection in vitro with a NT_50_ of 25 µg/mL. (**B**) Purified anti-ZIKV IgY did not enhance the ZIKV infection in vitro at 100 µg/mL. (**C**) Purified anti-ZIKV IgY did not enhance the DENV2 infection in vitro at 100 µg/mL, whereas 4G2 flavivirus IgG significantly enhanced the viral load. (**D**) Purified anti-ZIKV IgY did not enhance the ZIKV infection in vitro at any level tested, whereas 4G2 flavivirus IgG significantly enhanced the viral load at 1, 10, and 100 µg. **** *p* < 0.0001, ns = not significant.

Polyvalent anti-ZIKV IgY was purified from goose egg yolks that were tested for neutralization capacity. Concentrations ranging from 250 µg/mL to 2.5 µg/mL were utilized to determine the neutralization capacity of anti-ZIKV IgY in three independent experiments. Forty–50 PFUs of the Zika virus PRVABC59 were treated with anti-ZIKV IgY and then inoculated onto Vero cells in triplicate to determine the 50 percent neutralization titer (NT_50_). The concentration that yielded the NT_50_ was 25 µg/mL (Figure 2a). To determine if anti-ZIKV IgY induces ADE THP-1 cells, which can only become infected by the utilization of FcR-mediated endocytosis, the cells were challenged with treated stocks of ZIKV and DENV. The experimental positive control Pan-flavivirus IgG 4G2 antibody produced a productive viral replication cycle in THP-1 cells, yielding a significantly higher viral load compared to the untreated viral infection. The ZIKV-specific IgY did not enhance the viral burden at any of the concentrations tested (Figure 2b). Cross reactivity and potential enhancement of a secondary Dengue virus infection was also assessed as described above, and no viral enhancement was demonstrated in a DENV-2 infection of THP-1 cells. To confirm these findings, a lymphoblast cell line K-562, which are only permissive to ZIKV or DENV infection when the virus utilizes FcR-mediated endocytosis for entry, were infected in the same manner as the THP-1 cells. As demonstrated in the THP-1 cells, a productive viral infection in the cells treated with ZIKV-specific IgY was not achieved, while the 4G2 antibody produced robust viral infection. 

### 3.3. In Vivo Efficacy of ZIKV-Specific IgY

**Figure 3:**
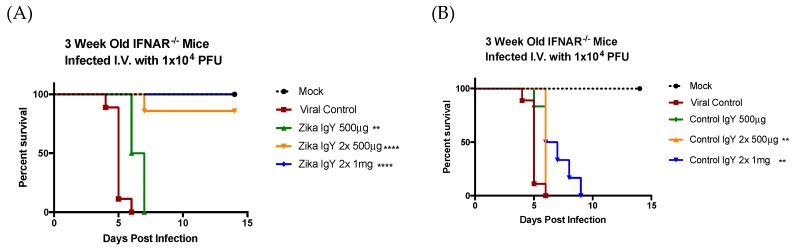

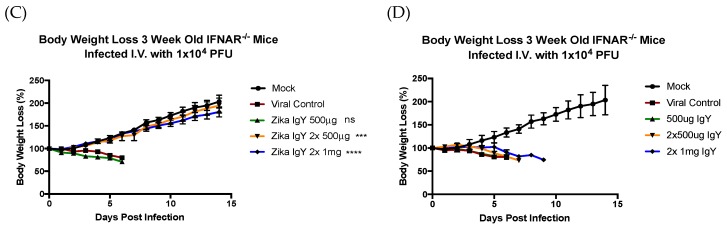
The therapeutic efficacy of anti-ZIKV IgY in vivo: Three-week-old IFNAR^−/−^ mice were administered a lethal dose of ZIKV intravenously (i.v.) in 100 µL total volume. At 24 and 48 h postinfection, the mice were immunized i.p. with the indicated amount of antibody in a volume of 100 µL. The control mice were treated with 100 uL of PBS. (**A**,**B**) The survival outcome of the treatments and (**C**,**D**) the weights were monitored daily until day 14 or until morbid. The weights were reported as a percent of the starting weight; the *p*-values are denoted when comparing the groups to the viral control. ** *p* < 0.01, *** *p* < 0.005, and **** *p* < 0.0001.

**Figure 4:**
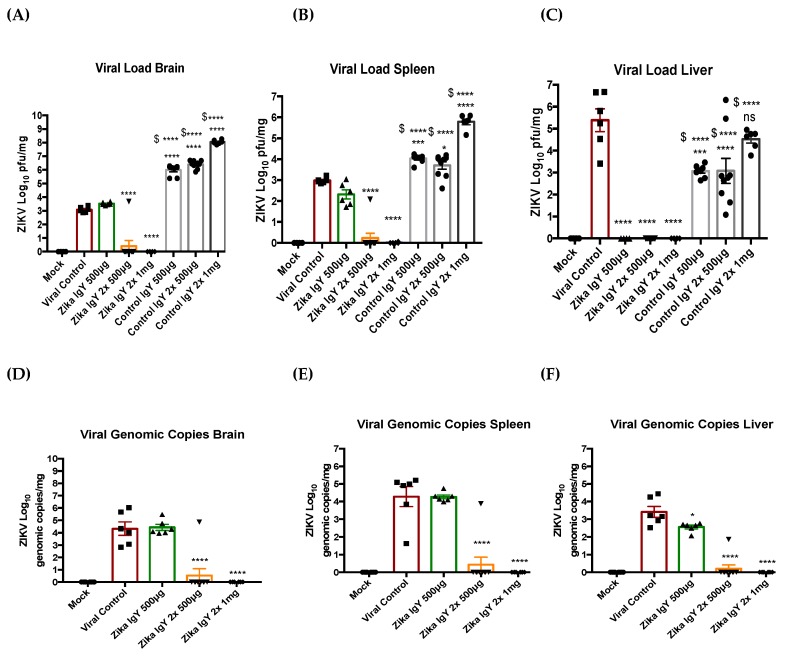
The viral load reduction upon treatment with anti-ZIKV IgY: (**A**–**C**) The infectious viral load was quantified in the brain (**A**), spleen (**B**), and liver (**C**) via a plaque assay. (**D**–**F**) The viral genomic copy number was quantified in the brain (**D**), spleen (**E**), and liver (**F**) via RT-qPCR. The viral load of the treated mice was compared to the viral control group. * *p* < 0.05, ** *p* < 0.01, *** *p* < 0.005, **** *p* < 0.0001, and $ is the comparison to the matching ZIKV-specific IgY group.

IFNAR^−/−^ mice were challenged with a lethal dose of ZIKV PRVABC59 (1 × 10^4^ PFUs), and the therapeutic potential of ZIKV-specific IgY was determined (Figure 3). The results of four independent experiments using a lethal dose of 1 × 10^4^ PFUs were combined. The mice receiving only PBS as a negative control succumbed to infection by 6 days postinfection. An initial dose of 500 µg of ZIKV-specific IgY (*n* = 6) 24 h postinfection significantly extended the lifespan but did not allow for recovery from the infection, and all mice succumbed to infection by day 7. Five hundred µg of ZIKV-specific IgY (*n* = 9) administered 24 and 48 h postinfection led to an 85% therapeutic efficacy. This dose significantly reduced the infectious viral load and genomic viral copies in the brain, liver, and spleen (Figure 4). We achieved a 100% therapeutic efficacy with 1 mg of ZIKV-specific IgY (*n* = 6) administered 24 and 48 h postinfection. This regimen resulted in a sterilizing immunity in all mice measured by the infectious viral load and viral genomic copies (Figure 4). Naïve IgY was used as a control; one dose of 500 µg (*n* = 8), two doses of 500 µg (*n* = 9), and 1 mg (*n* = 6) extended the mice’s lifespan, but no subjects survived the challenge. The resulting viral titers of the naïve IgY-treated mice were significantly increased compared to the challenge control group and the corresponding ZIKV-specific IgY dose, indicating the importance of priming the goose system with the viral antigen, as naïve IgY appears to enhance viral replication, specifically in the brain, by an unknown mechanism. 

### 3.4. ZIKV-IgY Epitope Mapping

**Figure 5:**
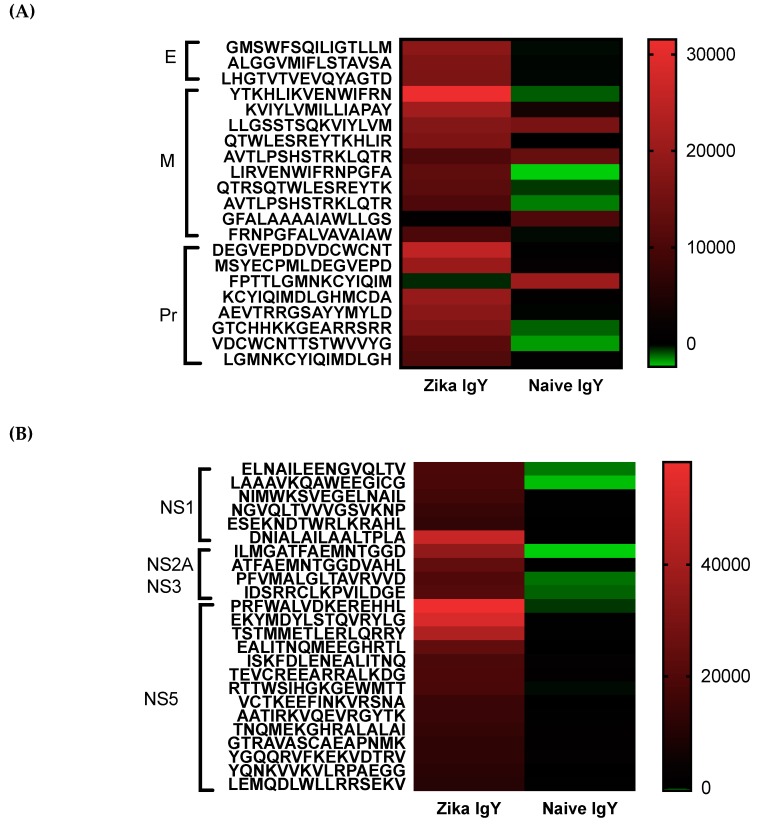
An epitope map of the structural and nonstructural genes: (**A**) A heat map of the structural epitopes of the Zika virus recognized by Zika-specific IgY and naïve IgY and (**B**) a heat map of the non-structural epitopes of the Zika virus recognized by Zika-specific IgY and naïve IgY. The strength of binding is indicated on a colorimetric scale with red being a strong binding affinity and green being no binding.

Epitope mapping was implemented to determine the specificity of the ZIKV-specific IgY. A pan-flavivirus microarray displaying overlapping linear epitopes of envelope (E); membrane (M); peptide pr (PR) capsid protein (C); nonstructural proteins 1, 2, 3, and 5 (NS1, NS2A, NS3, and NS5) reactivity was compared to the negative-control peptide mean fluorescent intensity average. Unbiased heat maps were generated in order to compare ZIKV-specific IgY to control naïve IgY (Figure 5). A majority of epitopes recognized by ZIKV-specific IgY were centralized on the M and NS5 protein. ZIKV-specific IgY did recognize a known neutralizing epitope on the E protein and a novel highly conserved NS1 epitope.

## 4. Discussion

The results reported herein show that anti-ZIKV IgY purified from goose egg yolk has the ability to neutralize the ZIKV infection in vitro and in vivo. We observed a therapeutic efficacy with 1 mg of anti-ZIKV IgY administered 24 and 48 h postinfection in a lethal IFNAR^−/−^ mouse model. Our results indicated that naïve IgY was able to partially delay death in a lethally infected mouse but that all mice immunized with naïve IgY succumbed to infection. This is in contrast to previous data from our lab demonstrating a nonspecific protection in a lethal DENV2 mouse infection model [15]. This may be due to the unique structural features of ZIKV; the mature virion is more thermally stable, allowing for less a dynamic rearrangement, making the fusion loop on the envelope protein more difficult to access [29]. As indicated in Figure 1, there is a full-length and alternatively spliced form of IgY. All experiments performed here utilized both the full-length and alternatively spliced forms of IgY in our purified product. In the future, these distinct forms will be separated and the neutralization capacity of each will be assessed. 

Epitope mapping was implemented to determine the specificity of the ZIKV-specific IgY and to identify potential broad neutralizing epitopes. A pan-flavivirus microarray displaying overlapping linear epitopes of envelope (E); membrane (M); peptide pr (PR) capsid protein (C); nonstructural proteins 1, 2, 3, and 5 (NS1, NS2A, NS3, and NS5) was compared to negative control peptides. The mean fluorescent intensity of the negative control peptides was used to remove the fluorescent signal from nonspecific binding. Unbiased heat maps were generated in order to compare ZIKV-specific IgY to control naïve IgY (Figure 5). We found that the peptide LHGTVTVEVQYAGTD of the IgG III-like domain of the E protein was strongly recognized, which is critical for cell receptor binding as well as endosomal fusion. A NS1 epitope was a highly recognized peptide, ESEKNDTWRLKRAHL, that is conserved across multiple strains of ZIKV. This epitope has not been previously identified in other antibody screening studies. Future studies are planned to isolate these epitope-specific IgY populations and to determine their ability to neutralize ZIKV. At this time, the oligoclonal ZIKV-specific IgY preparation effective dose is quite high for therapeutic use. By enriching the IgY preparation with known neutralizing epitopes, we can drastically decrease the number of antibodies needed to induce efficacy. Studies will be done in the future to lower the dosage in this manner. We measured the potential cross-reactivity of the polyclonal Zika-specific IgY with other flaviviruses via a microarray analysis. We found a significant cross-reactivity with the Dengue virus, Yellow Fever virus, and West Nile virus (Supplemental Figure S1). The epitopes corresponding to the number on the microarray are displayed in Supplemental Table S1. The potential for the cross neutralization of these three viruses will be tested in the future. 

Since the production of antivirals and vaccines may be difficult, alternative therapies must be explored for emerging pathogens such as ZIKV. Avian IgY and egg powder have been declared by the United States Code of Federal Regulation as generally recognized as safe (GRAS) [30]. The therapeutic potential of polyclonal avian antibodies has been demonstrated for a number of pathogens. Gastrointestinal infections such as enterotoxic *Escherichia coli*, *Salmonellal* spp, Rotavirus, and *Helicobacter pylori* have been successfully treated in mice and humans by utilizing IgY [31]. IgY has also been implemented in Sweden as an orphan drug for the prevention of *Pseudomonas aeruginosa* in cystic fibrosis patients [30]. We recognize that translating anti-ZIKV IgY to a human therapy will encounter challenges. It has been previously demonstrated that pigs and mice will develop a humoral response to avian IgY, notably the subclass IgG [32,33]. Unpublished data from our lab demonstrated weekly IgY injections begin to produce anti-IgY IgG in the third to fourth weeks of immunizations. Purified IgY is not known to have the ability to induce an allergic reaction in humans. What has been demonstrated is that raw egg yolk, purified IgY, and alternatively spliced IgY did not induce an IgE response in mice. Egg allergies are important to consider when implementing IgY as a therapy in case residual egg yolk lipids still remain in the purified product. Future allergenicity studies will need to be conducted to definitively determine if purified IgY induces an allergic response or not.

At the present, there are no FDA-approved vaccines or therapies for the ZIKV infection. Multiple platforms, live attenuated, nucleic acid-, vector-, and peptide-based, have been employed to develop a safe efficacious vaccine for ZIKV [34,35,36]. A major conundrum for the development of a ZIKV vaccine is the risk of inducing ADE by a subsequent DENV infection. Settler et al. demonstrated that cross-reactive ZIKV antibodies have the ability to enhance a DENV infection [13]. The severity of developing a therapy without properly assessing its ability to induce ADE was demonstrated by the deployment of the tetravalent dengue vaccine that resulted in an increased hospitalization in younger and serum naïve individuals [37]. This study emphasized the need to produce vaccine and therapies for flaviviruses that do not induce ADE, such as the case of anti-ZIKV IgY demonstrated here. IgY is similar to aglycosylated IgG, as IgY does not bind to FcγR. IgY is advantageous because it does not require any genetic modification or postproduction processing to prevent enhancement.

Teratogenic pathology induced by ZIKV when a mother is infected and the infection spreads to the fetus is a major concern in the recent outbreak. Due to the causal relationship between ZIKV and the development of microcephaly led the WHO to declare ZIKV as a public health emergence and to expedite therapies needed to combat the ZIKV infection. The mechanism by which IgY neutralizes in a mammalian host is still under study. The mammalian homologous to the FcYR, which is used to transport IgY across the egg yolk sac, are the phospholipase A2 receptor and mannose receptor [38]. Each of these receptors are expressed on the mammalian placenta, potentially indicating a route in which IgY could transverse the placenta and protect a developing fetus [39,40,41,42]. Taking this statement into account, future studies will examine the therapeutic efficacy of immunizing dams with anti-ZIKV IgY and will determine the amount of cross-placental transmission and the prevention of viremia of the fetus. 

In summary, our studies are the first to comprehensively characterize the therapeutic potential of anti-ZIKV IgY and to demonstrate that IgY does not induce ADE of ZIKV in vitro. Our results indicate that utilizing a polyethylene glycol extraction method obtained a highly pure IgY product. Our purified anti-ZIKV IgY was able to inhibit the ZIKV infection in vitro and did not induce ADE at multiple concentrations. Anti-ZIKV IgY therapeutically administered to mice protected against a lethal ZIKV challenge. Epitope mapping demonstrated the recognition of known neutralizing E epitopes as well as novel NS1 epitopes unique to avians. Thus, our study has addressed an important aspect of the ZIKV infection, i.e.; a safe and effective immunotherapy that may be potentially utilized in an epidemic outbreak of ZIKV. 

## Supplementary Materials

The following are available online at https://www.mdpi.com/1999-4915/11/3/301/s1, Figure S1. The microarray profile of cross-reactive epitopes to other flaviviruses. Table S1. The amino acid sequences corresponding to the epitope number in Figure S1.
