# Supplementary material for: Zika Virus-Specific IgY Results Are Therapeutic Following a Lethal Zika Virus Challenge without Inducing Antibody-Dependent Enhancement
Source: Viruses. 2019 Mar 26;11(3):301. doi: 10.3390/v11030301 (PMC6466411; doi:10.3390/v11030301)
Supplement: Supplementary file 1 [file viruses-11-00301-s001.zip › Zika IgY Supplemental /Supplemental Fig 1.docx]

**Supplemental Fig 1: Epitope mapping of structural and non-structural genes.** **A.)** A heat map of structural epitopes of DENV recognized by Zika specific IgY and naïve IgY. **B.)** A heat map of non-structural epitopes of DENV recognized by Zika specific IgY and naïve IgY. **C.)** A heat map of structural epitopes of WENV recognized by Zika specific IgY and naïve IgY. **D.)** A heat map of non-structural epitopes of WENV recognized by Zika specific IgY and naïve IgY. **E.)** A heat map of structural epitopes of YFV recognized by Zika specific IgY and naïve IgY. **F.)** A heat map of non-structural epitopes of YFV recognized by Zika specific IgY and naïve IgY The strength of binding is indicated on a colorimetric scale with red being strong binding affinity and green being no binding.

C.)

E.)

F.)

B.)

D.)

A.)
