# Supplementary material for: Zika Virus-Specific IgY Results Are Therapeutic Following a Lethal Zika Virus Challenge without Inducing Antibody-Dependent Enhancement
Source: Viruses. 2019 Mar 26;11(3):301. doi: 10.3390/v11030301 (PMC6466411; doi:10.3390/v11030301)
Supplement: Supplementary file 1 [file viruses-11-00301-s001.zip › Zika IgY Supplemental /Supplemental Table 1.docx]

| **Position** | **Dengue Structural Proteins** | | **Dengue Non-Structural Proteins** | |
| --- | --- | --- | --- | --- |
| 1 | LEHGSCVTTMAKNKP | E | GTTVVVDEHCGNRGP | NS1 |
| 2 | TAWDFGSLGGVFTSI | E | KAKIIGADVQNTTFI | NS1 |
| 3 | QGEPSLNEEQDKRFV | E | ASAIQKAHEEGICGI | NS1 |
| 4 | PLPWLPGADTQGSNW | E | FIIDGPNTPECPSAS | NS1 |
| 5 | TTESRCPTQGEPSLN | E | MEIRPLSEKEENMVK | NS1 |
| 6 | QKETLVTFKNPHAKK | E | GTTVVITENCGTRGP | NS1 |
| 7 | LTLDCEPRSGIDFNE | E | MEIRPLSEKEENMVK | NS1 |
| 8 | NIEAEPPFGESYIVI | E | LENLMWKQITPELNH | NS1 |
| 9 | MAAILAYTIGTTHFQ | M | EKYQLAVTIMAILCV | NS2A |
| 10 | MAYMIGQTGIQRTVF | M | IAGVFFTFVLLLSGQ | NS2A |
| 11 | VEKVETWALRHPGFT | M | NQILEENVEVEIWTK | NS3 |
| 12 | SVALVPHVGMGLETR | M | FRKRKLTIMDLHPGA | NS3 |
| 13 | LFLAHAIGTSITQKG | M | TDGPERVILAGPIPV | NS3 |
| 14 | IGQTGIQRTVFFVLM | M | PGSRDPFPQSNAPIM | NS3 |
| 15 | SSEGAWKHAQRIETW | M | DAFPQSNAPIQDEER | NS3 |
| 16 | VPHVGMGLETRTETW | M | TATPPGATDPFPQSN | NS3 |
| 17 | FKTEDGVNMCTLMAM | Peptide_pr | EDMLTVWNRVWIEDN | NS5 |
| 18 | IDCWCNLTSTWVMYG | Peptide_pr | GEGLHRLGYILEEID | NS5 |
| 19 | EPRMIVGKNERGKSL | Peptide_pr | DVFYLPPEKCDTLLC | NS5 |
| 20 | CNLTSTWVMYGTCTQ | Peptide_pr | LGEKWKSRLNALGKS | NS5 |
| 21 | CPLLVNTEPEDIDCW | Peptide_pr | YFHRRDLRLASNAIC | NS5 |
| 22 | FKTTEGINKCTLIAM | Peptide_pr | GDDCVVKPLDERFGT | NS5 |
| 23 | RWGSFKKNGAIKVLR | C | HGSYEVKATGSASSM | NS5 |
| 24 | AFITFLRVLSIPPTA | C | KKVTEVKGYTKGGPG | NS5 |
| 25 | RKEIGRMLNILNGRK | C | DVFFIPPEKCDTLLC | NS5 |
| 26 | PTAGILKRWGQLKKN | C | YILRDVSKKEGGAMY | NS5 |
| 27 | LIGFRKEIGRMLNIL | C | YFHRRDLRLASMAIC | NS5 |
| 28 | LAKRFSKGLLSGQGP | C | QVRSLIGNEEYTDYM | NS5 |
| 29 | MNQRKKVVRPPFNML | C | QEEQGWTSASEAVND | NS5 |
| 30 |  |  | YILEEIDKKDGDLMY | NS5 |
| 31 |  |  | KPTEQVDTLLCDIGE | NS5 |
| 32 |  |  | DLENPHLPEKKITQW | NS5 |
| 33 |  |  | LVKLHSGKDVFFIPP | NS5 |
| 34 |  |  | CVVKPLDDRFASALT | NS5 |
| 35 |  |  | SVWNRVWIEENPWME | NS5 |
| 36 |  |  | SRKEFDLYKKSGITE | NS5 |
| 37 |  |  | DVSKKEGGAMYADDT | NS5 |

| **Position** | **West Nile Structural Proteins** | | **West Nile Non-Structural Proteins** | |
| --- | --- | --- | --- | --- |
| 1 | VVFAILLLLVAPAYS | E | ECPTQNRAWNSLEVE | NS1 |
| 2 | IDVKMMNMEAANLAE | E | NTLLKENGVDLSVVV | NS1 |
| 3 | EFEEPHATKQSVIAL | E | TRTTTESGKLITDWC | NS1 |
| 4 | SLTVQTHGESTLANK | M | CDSKIIGTAVKNNMA | NS1 |
| 5 | QTHGESTLANKKGAW | M | IIPITLAGPRSNHNR | NS1 |
| 6 | ESTLANKKGAWMDST | M | TPQGLAKIIQKAHKE | NS1 |
| 7 | KVMMTVNATDVTDVI | Peptide_pr | VSRLEHQMWEAVKDE | NS1 |
| 8 | CEDTITYECPVLAAG | Peptide_pr | VVDGPETKECPTQNR | NS1 |
| 9 | ITYECPVLAAGNDPE | Peptide_pr | EIDFDYCPGTTVTIS | NS1 |
| 10 | DVITIPTAAGKNLCI | Peptide_pr | ASTGVFNPMILAAGL | NS2A |
| 11 | ITYECPVLSAGNDPE | Peptide_pr | LLVVFLATQEVLRKR | NS2A |
| 12 | CEDTITYECPVLAAG | Peptide_pr | KEKRSSAAKKKGACL | NS2A |
| 13 | PEDIDCWCTKSSVYV | Peptide_pr | FLELLRTADLPVWLA | NS3 |
| 14 | CPVLAAGNDPEDIDC | Peptide_pr | PGAGKTRRILPQIIK | NS3 |
| 15 | KSRAVNMLKRGMPRV | C | NPSQVGDEYCYGGHT | NS3 |
| 16 |  |  | QRRGRIGRNPSQVGD | NS3 |
| 17 |  |  | WLAYKVAAAGISYHD | NS3 |
| 18 |  |  | SAIVQGERMEEPAPA | NS3 |
| 19 |  |  | GRTTWSIHAGGEWMT | NS5 |
| 20 |  |  | VVPCRGQDELIGRAR | NS5 |
| 21 |  |  | RTWNYHGSYDVKPTG | NS5 |
| 22 |  |  | KDIQEWKPSTGWYDW | NS5 |
| 23 |  |  | AENIHVAINQVRSVI | NS5 |
| 24 |  |  | ENEWMEDKTPVERWS | NS5 |
| 25 |  |  | STGWYDWQQVPFCSN | NS5 |
| 26 |  |  | VSRASGNVVHSVNMT | NS5 |
| 27 |  |  | EEQNQWRSAREAVED | NS5 |
| 28 |  |  | KNSGGGVEGLGLQKL | NS5 |
| 29 |  |  | GKREDIWCGSLIGTR | NS5 |
| 30 |  |  | HRRLARAIIELTYRH | NS5 |
| 31 |  |  | IERLRREYSSTWHHD | NS5 |
| 32 |  |  | YGWNIVTMKSGVDVF | NS5 |

| **Position** | **Yellow Fever Structural Proteins** | | **Yellow Fever Non-Structural Proteins** | |
| --- | --- | --- | --- | --- |
| 1 | KGTTYGVCSKAFKFL | E | WCCRSCTMPPVSFHG | NS1 |
| 2 | KVIMGVVLIWVGINT | E | AAVNGKKSAHGSPTF | NS1 |
| 3 | FGGLSWITKVIMGVV | E | AINFSKRELKCGDGI | NS1 |
| 4 | TALTIAYLVGSNMTQ | M | NLVFSPGRKNGSFII | NS1 |
| 5 | ERWLVRNPFFAVTAL | M | WKLEGRWDGEEEVQL | NS3 |
| 6 | ERWLVRNPFFAATAL | M | GKKLVPSWASVKEDL | NS3 |
| 7 | VRNPFFAVTALTIAY | M | WQVAKAGLKTNDRKW | NS3 |
| 8 | LQKIERWLVRNPFFA | M | EPTRVVNWEVIIMDE | NS3 |
| 9 | VTLVRRNRWLLLNVT | Peptide_pr | VTRMAMTDTTPFGQQ | NS5 |
| 10 | YGKCDSAGRSRRSRR | Peptide_pr | WHYCGSYVTRTSGSA | NS5 |
| 11 | YGKCDSAGRSRRSRR | Peptide_pr | KVDTRAKDPPAGTRK | NS5 |
| 12 | CWCYGVENVRVAYGK | Peptide_pr | RTTWSIHGKGEWMTT | NS5 |
| 13 | REEPDDIDCWCYGVE | Peptide_pr | WRDVPYLTKRQDKLC | NS5 |
| 14 | VTLVRKNRWLLLNVT | Peptide_pr | VSPGNGWMIKETACL | NS5 |
| 15 | TLVRRNRWLLLNVTS | Peptide_pr | GGVEGIGLQYLGYVI | NS5 |
| 16 | FSVGTGNCTTNILEA | Peptide_pr | KGPLDRAAIEERVER | NS5 |
| 17 | GLAVLRKVKRVVASL | C | CVVRPIDDRFGLALS | NS5 |
| 18 | QKTKQIGNRPGPSRG | C | QDKTMVKEWRDVPYL | NS5 |
| 19 | WKMLDPRQGLAVLRK | C | GKVDTGVAVSRGTAK | NS5 |
| 20 | WKMLDPRQGLAVLRK | C | GKAYMDVISRRDQRG | NS5 |
| 21 | FNILTGKKITAHLKR | C |  |  |

**Supplemental Table 1:** Corresponding epitopes to the heat maps of structural and non-structural proteins of DENV, WENV, and YFV recognized by anti-ZIKV IgY and naïve IgY.
